# Mechanical Characterization and Impact Damage Assessment of Hybrid Three-Dimensional Five-Directional Composites

**DOI:** 10.3390/polym11091395

**Published:** 2019-08-24

**Authors:** Liwei Wu, Wei Wang, Qian Jiang, Chunjie Xiang, Ching-Wen Lou

**Affiliations:** 1 Tianjin and Ministry of Education Key Laboratory for Advanced Textile Composite Materials, Tianjin Polytechnic University, Tianjin 300387, China; 2 Innovation Platform of Intelligent and Energy-Saving Textiles, School of Textile Science and Engineering, Tianjin Polytechnic University, Tianjin 300387, China; 3 Department of Bioinformatics and Medical Engineering, Asia University, Taichung 41354, Taiwan; 4 Department of Medical Research, China Medical University Hospital, China Medical University, Taichung 40402, Taiwan; 5 College of Textile and Clothing, Qingdao University, Shandong 266071, China

**Keywords:** hybrid composite, carbon fiber, aramid fiber, low-velocity impact

## Abstract

The effects of braided architecture and co-braided hybrid structure on low-velocity response of carbon-aramid hybrid three-dimensional five-directional (3D5d) braided composites were experimentally investigated in this study. Low-velocity impact was conducted on two types of hybridization and one pure carbon fiber braided reinforced composites under three velocities. Damage morphologies after low-velocity impact were detected by microscopy and ultrasonic nondestructive testing. Interior damages of composites were highly dependent on yarn type and alignment. Impact damage tolerance was introduced to evaluate the ductility of hybrid composites. Maximum impact load and toughness changed with impact velocity and constituent materials of the composites. The composite with aramid fiber as axial yarn and carbon fiber as braiding yarn showed the best impact resistance due to the synergistic effect of both materials. Wavelet transform was applied in frequency and time domain analyses to reflect the failure mode and mechanism of hybrid 3D5d braided composites. Aramid fibers were used either as axial yarns or braiding yarns, aiding in the effective decrease in the level of initial damage. In particular, when used as axial yarns, aramid fibers effectively mitigate the level of damage during damage evolution.

## 1. Introduction

As required by high-performance materials in aircraft and aerospace applications, three-dimensional (3D) braided composites show unique advantages against the delamination of traditional laminated composites due to the highly integrated structure of reinforced fiber network [1,2]. 3D braided composites can achieve fabrication of “net-shape” complex structural components, which are suitable for designing material structure performance-integrated composites [3,4]. Compared with the basic 3D braided structure, the 3D five-directional (3D5d) braided structure improved the in-plane mechanical properties and structural integrity with the insertion of axial yarns [5]. The spatially distributed fiber bears loading through thickness direction, thus improving the mechanical property of composites, especially the impact property. 

Impact loading has become one of the most important parameters when measuring the rationality and reliability of structural composite design [6]. Previous research results showed that impact damage is the most serious damage caused by the usage of fiber-reinforced composites [7]. Especially after low-speed impact, large-area delamination, matrix cracking, interface debonding, and fiber fracture occur on the surface and inside of composites [8,9], decreasing the strength and stiffness of the material and inevitably affecting the load carrying capacity and service life of composites. Therefore, the composite impact property has attracted considerable research attention. Moure et al. [10] analyzed the behavior of aramid composite plates subjected to low and medium impact energies (20 J < *E* < 600 J) and showed a greater energy absorption capacity in thin plates than in thick plates for low impact energy values. Aswani et al. tested the energy absorption capacity of three-dimensional angular interlocking fabrics of aramid, basalt and arami/basalt under low velocity impact, the results show that the hybrid composite has the highest energy absorption capacity [11]. Hufenbach et al. studied the strain rate-dependent material properties and failure behavior of composites with hybrid fiber reinforcements and concluded that by intelligent combination of different fiber materials in the textile preform, the stiffness, strength, and crash-worthiness of 3D textile preforms can be adjusted [12]. Thus, hybrid 3D textile preforms are excellent candidates for use as impact and crash components of innovative lightweight structures for the aircraft and vehicle industry and for mechanical engineering applications.

Hybridization characteristics were first reported in 1972 [13]. The advantage of hybridization is the compensation of the disadvantage of one type of fiber by the other types. Carbon fibers have been reported to offer excellent mechanical properties but low impact resistance due to their rigidity and fragility [14]. Carbon fibers were hybridized with other fibers as reinforcement to improve their impact property. Wan et al. [15,16] tested the flexural, shear, and impact properties of intrayarn hybrid 3D braided composites to determine the hybrid effects of carbon and Kevlar fibers. They observed that the carbon–aramid hybrid composites can be developed with strength and toughness that are far superior to those of their individual reinforced composites. Bunea et al. [17] investigated the influence of matrix properties, the number of carbon and aramid layers, and fiber orientation on the low-velocity-impact behavior of fabric-reinforced hybrid composites. However, few investigations focused on the failure mechanism of 3D5d braided composites, which are subjected to low-velocity-impact loading.

This research aimed to determine the effect of braided architecture and hybrid structure on the impact response of carbon-aramid hybrid 3D5d braided composites under different impact velocities. Damage morphologies were detected by ultrasonic nondestructive testing, and impact damage toleraquency domain and time domain analyses to reflect the failure mode and mechanism of hybrid 3D5d braided composites. The extensive results provide an indication for an improved design of 3D reinforcement with respect to specific impact conditions and serve as basis for the development of material and structural behavior failure of 3D5d braided composite under impact loads.

## 2. Materials and Experiments

### 2.1. Materials

Carbon fiber tows (T300-3K, Toray®, Tokyo, Japan) with tow size of 200 tex × 2 tex, aramid fiber (Kevlar49, DuPont®, DE, USA) with tow size of 158 tex × 2 tex and epoxy resin (TDE-86, Jingdong®, Tianjin, China) were used for fabricating 3D5d hybrid braided composites. Table 1 shows the specific properties of raw materials.

### 2.2. Sample Preparation

**Figure 1:**
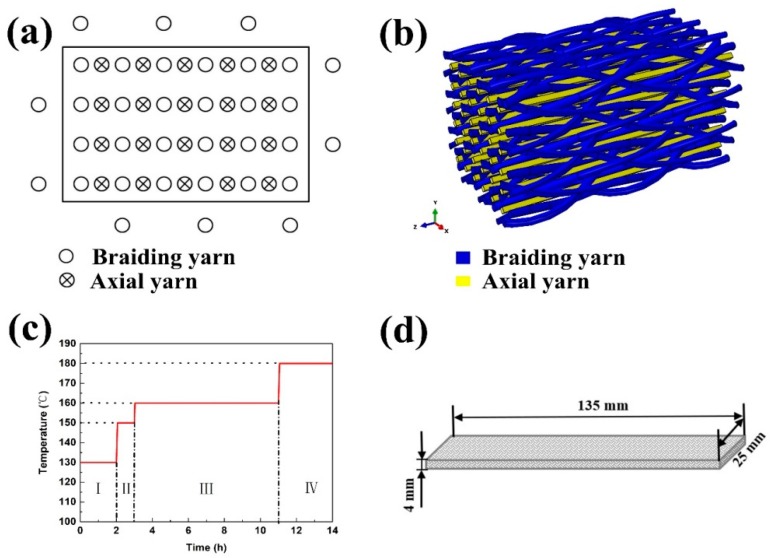
Sample preparation diagrams, (**a**) braiding process; (**b**) braiding macroscopic structure; (**c**) curing temperature program; (**d**) composite dimension.

A four-step 1 × 1 braiding technique was applied to braid the 3D5d structure with a rectangular cross section. Different from the traditional 3D4d structure, 3D5d has axial yarns aligned along the longitudinal direction of braided preform. The sample specifications are shown in Table 2. The composites were prepared by resin transfer molding (RTM). Under the condition of injection pressure of 0.2~0.3 MPa and a temperature of 130 °C, the resin was injected into the sealing mold with prefabricated parts. After the resin was completely impregnated into the preforms, they were cured in an oven. The preparation process and sample dimension are shown in Figure 1. The specific specifications of three kinds of composites are shown in Table 2, where C represents carbon fiber, K represents aramid, a represents a yarn, and b represents a woven yarn.

### 2.3. Tests

#### 2.3.1. Quasi-Static Bending Tests

The three-point bending loading test was carried out by SHIMADZU AG-250KNE universal material machine using ASTM D7264/D7264M-07 standard, the loading speed was 2 mm/min, the room temperature was 20–28 °C and the relative humidity was not more than 50%.

#### 2.3.2. Dynamic Impact Test

As specified in the ASTM D7136/D7136M-15 standard, impact test was performed at impact velocities of 2, 4, and 6 m/s at room temperatures of 20–28 °C and a relative humidity of >50% using an Instron Dynatup 9250 HV. The falling weight of mass equaled 6.5 kg, and a hemispherical striker contained a tip measuring 12.7 mm in diameter. Impact velocity was marked as the subscript of sample type, i.e., CaCb2 indicates that the impact velocity is 2 m/s.

#### 2.3.3. Ultrasonic Non-Destructive Testing

Employing the FirstMap ultrasonic scanning detection system, the 3.5 MHz ultrasonic sound source was used to scan the sample with a probe coated with a coupling agent. Due to the compositional inconsistency in hybrid composite, the conventional frequency of 5 MHz for single component composite is not proper in this study. Inspired by ref. [18], 3.5 MHz ultrasonic sound source was chosen. The probe demonstrated both ultrasonic emission and receiving functions when encountering different material interfaces. The energy ultrasound was reflected with the C-scan result being outputted by the computer, the results of which showed the internal damage distribution. 

## 3. Results and Discussions

### 3.1. Quasi-Static Mechanical Property

**Figure 2:**
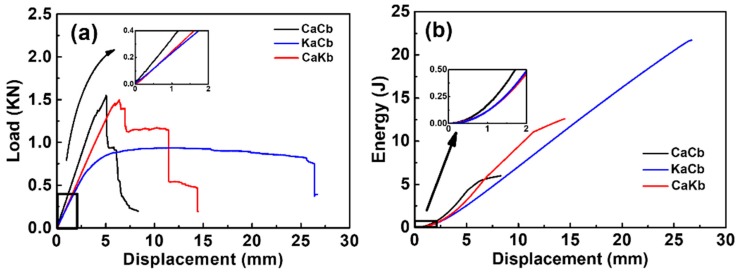
Three-dimensional five-directional (3D5d) braiding composites under quasi-static bending test. (**a**) Load versus displacement curve; (**b**) energy absorption versus displacement curve.

The quasi-static bending test was conducted to provide a basis on the dynamic evaluation of 3D5d hybrid braiding composites. Load-displacement curves of three types of 3D5d braiding composite demonstrate obvious difference under quasi-static bending test, as shown in Figure 2a. The flexural modulus and strength of CaCb series are the highest as 60.5 GPa and 585 MPa, respectively. KaCb and CaKb have similar flexural modulus of 34.85 and 34.92 GPa, which is interesting and probably due to the combined effect the fiber position and its volume fraction, that is; axial yarns bear the majority of load when subjected to bending but the volume fraction is lower; braiding yarn has higher volume fraction, however, they bear lower loading, thus leading to the compromised effect. A homogenization algorithm for composite [19] was applied to calculate the stiffness matrices of KaCb and CaKb to make sure the similar flexural modulus is reasonable, shown in Equations (1) and (2). The curves reveal that the 3D5d braided composite composed of pure carbon fiber was brittle, whose strength suddenly dropped to zero when the load reached the maximum value, completely in conformity with linear elastic characteristics. Comparatively, due to the lower modulus of aramid fiber than carbon fiber, the bending modulus and strength of the 3D5d hybrid braiding composites (CaKb and KaCb series) were both lower than CaCb series with deflections being larger. The CaKb series presented progressive damages at 76% and 36.7% of maximum load, while the KaCb series showed obvious ductile behavior with a long plateau to 25.6 mm. Meanwhile, the energy absorption can be calculated by the integral of the load versus displacement curve, as shown in Figure 2b. The energy absorption of hybrid series is higher than CaCb series, and the higher absorption energy further illustrates the toughness improvement brought by hybrid effect. KaCb series reaches a maximum of 21.6 J, which is 3.6 times higher than CaCb series.

(1) [C]KaCb=[28.08036.50986.50980006.50989.39714.64010006.50984.64019.3971000 0003.95590000006.14840 000006.1484]

(2) [C]CaKb= [28.76194.85284.85280004.85289.38164.22510004.85284.22519.3816000 0003.30800000004.43540 000004.4354]

### 3.2. Dynamic Impact Property

**Figure 3:**
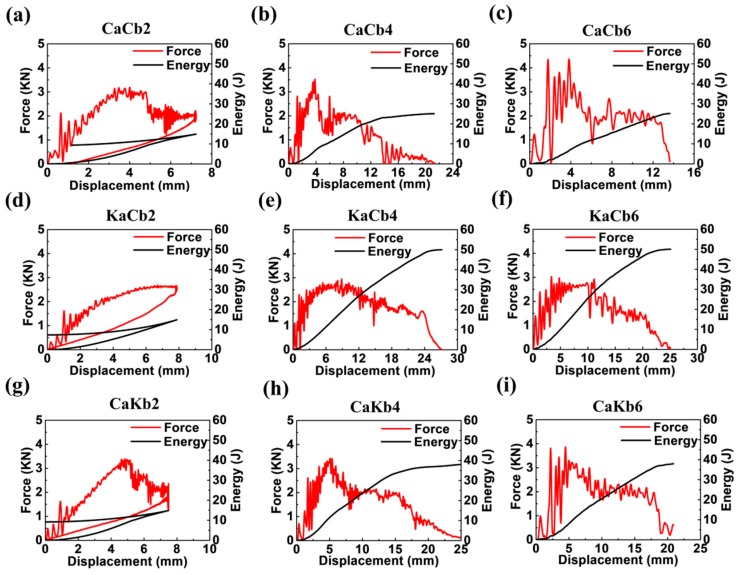
Load/energy-displacement curves of 3D5d braided composites. The three rows represent the sample types of (**a**–**c**) CaCb, (**d**–**f**) KaCb, and (**g**–**i**) CaKb, whereas the three columns represent the impact velocities of (a,d,g) 2 m/s, (b,e,h) 4 m/s, and (c,f,i) 6 m/s, respectively.

Figure 3 shows the load/energy-displacement curves of 3D5d braided composites in relation to impact velocity. CaCb2, KaCb2 and CaKb2 experience severe fluctuations at the initial stage, suggesting the formation of initial damage. When the load reaches the maximum, CaCb2 and CaKb2 decrease sharply and violent fluctuations occur, while KaCb2 maintain a steady value. Finally, the rebounds take place and the elastic release energy is transmitted to the impact head, forming the loops for three types of composites. When the impact velocity reaches 4 and 6 m/s, the loops disappear, revealing no rebound occurs and further proving the damage occurrence under higher impact velocities. However, KaCb4 and KaCb6 still show great advantages over other composites since they continue to bear load after the maximum load is achieved, effectively inhibiting the evolution of damage. From the phenomenon observed from Figure 3, it can be concluded that different axial yarns exhibit considerable influence on the impact response at maximum impact load. In particular, axial yarns consisting of aramid fibers significantly strengthen the impact resistance of composite, which is due to the higher ductility derive from different molecular structure between carbon fiber [20] and aramid fiber [21].

The absorption energy-displacement curves of CaCb2, KaCb2 and CaKb2 are firstly increased when external energy is transferred to the inside of the composite and leads to deformation and damage. Then the elastic deformation energy is released to the impact head, forming the energy loops. Eventually, CaCb2, KaCb2 and CaKb2 absorb energy of 10.0, 7.3 and 9.2 J, separately. Since the impact energy is 15 J, the elastic deformation energies of CaCb2, KaCb2 and CaKb2 are 5, 7.6 and 5.8 J, respectively. At an impact velocity of 4 m/s, the impact energy is calculated to be 61 J. The absorbed energy-displacement curves first go up and then reach the point at which damage dissipates most of the energy and the energy reaches maximum. CaCb4, KaCb4 and CaKb4 eventually absorb energy of 25, 50, and 37 J, respectively. Among them, KaCb4 absorbs the highest energy, which indicates that when served as the axial yarn, the aramid fibers effectively bear the load and absorb energy when impact is imparted. When the impact velocity reaches 6 m/s, although the impact energy is increased to 138 J, it is interesting to find out that the final energy absorption of CaCb6, KaCb6 and CaKb6 are consistent with those of 4 m/s, reflecting that maximum energy absorption for CaCb, KaCb and CaKb are around 25, 50, and 37 J when accounting into variation under different impact velocities. 

**Figure 4:**
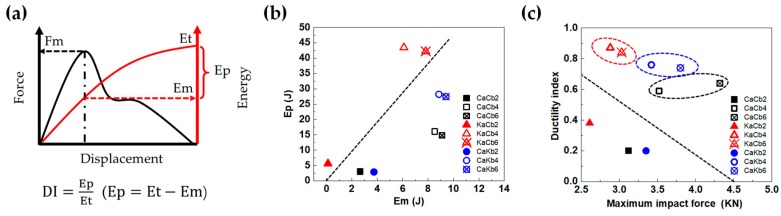
Ductility index calculation (**a**), energy distribution (**b**), and ductility index distribution at maximum impact load (**c**) of 3D5d braided composites.

To investigate the impact damage tolerance of 3D5d braided composites, the ductility index (DI) was adopted to illustrate the ductility performance of composites during the impact process. For most materials, the DI is defined as the energy absorption after the maximum load divided by the energy absorption to the maximum load [22]. However, for the hybrid composite, the failure could be initiated earlier than the maximum load point as shown in Figure 3. Therefore, it may be more meaningful to use total energy (*E*_t_) in calculating DI, as shown in Figure 4a, among which E_p_ represents propagation energy that can be calculated by the difference between total energy (*E*_t_) and energy absorption to the maximum load (*E*_m_). Figure 4b presents the energy distribution of 3D5d braided composites relating *E*_p_ and *E*_m_. Due to the high level of integration of 3D5d braided structure, the Ep value is mostly higher than *E*_m_ but to a different degree for all of the three types of composites. This result indicates that the 3D5d braided composites can effectively prevent crack propagation after impact. Dotted lines are plotted when *E*_p_ = 5*E*_m_, acting as the division line between high energy absorption region and low energy absorption region. A distinct difference in energy distribution is observed between the KaCb which occupies the above region of the dotted line, and CaCb and CaKb which are found below. At an impact velocity of 2 m/s, failure does not appear for KaCb2, revealed by *E*_m_ equal to zero, while CaCb2 and CaKb2 exhibit failure after maximum load, resulting in the lower *E*_p_/*E*_m_. Impact velocities of 4 and 6 m/s result in obvious jumps of E_p_ for three types of composites, however, KaCb still outperforms CaCb and CaKb and the latter two show comparable *E*_p_/*E*_m_ ratios, indicating that CaCb and CaKb dissipate energy with same pattern under higher impact energy. Consistent with what has been described in Figure 3, the *E*_t_ becomes constant under impact velocities of 4 and 6 m/s, therefore, a dynamic balance between *E*_p_ and *E*_m_ can be observed from Figure 4b.

Figure 4c shows the ductility index distribution under maximum impact force; the larger the DI value, the better toughness the composite has. It can be clearly seen that at 2 m/s impact velocity, three composites have both low DI and maximum impact force due to the incomplete damage. With the increase of impact velocity, the impact responses of composites show remarkable improvement on composite toughness. Symbols above the dotted line exhibit impact energy higher than failure energy threshold, and those below it shows impact energy lower than failure energy threshold. Hence, two phenomena are notable: (1) KaCb exhibited the highest DI and lowest maximum impact load, but the opposite is the case for CaCb. (2) Increasing the impact velocity from 4 to 6 m/s renders differences in the three composite types. CaCb demonstrates considerable maximum impact load but the same DI. KaCb is composed of aramid fibers as axial yarns and exhibits a slight increase in DI. By contrast, CaKb possesses aramid fibers as braiding yarns and shows a slight decrease in DI. Given that axial yarns demonstrate remarkable influence on the impact resistance of the composites when impact velocity is increased, the mechanical properties of aramid fibers are affected more by strain rate compared with those of carbon fibers. Therefore, the DI of KaCb and CaKb changed accordingly. The relation between maximum impact load and DI is based on the impact velocity and constituents of the composites. 

### 3.3. Damage Morphology Analyses

**Figure 5:**
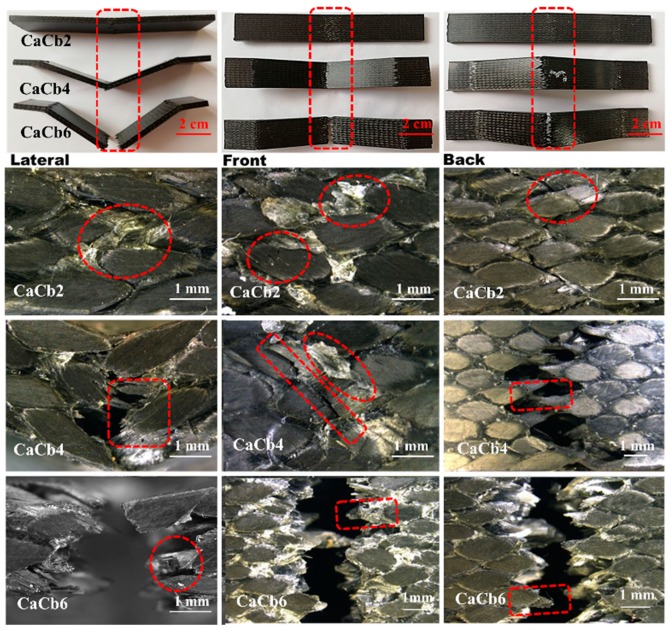
Damage morphology of 3D5d braided composite of CaCb series after impact with impact velocity of 2, 4 and 6 m/s from lateral, front and back views; subscripts 2, 4, 6 represent impact velocity 2, 4 and 6 m/s.

Figure 5 shows damage morphologies of 3D5d braided composite of CaCb series after impact from lateral, front and back views. It can be seen, that after the 2 m/s impact, a slight damage appears with mostly a resin crack which exhibits white spots in red dotted circles. With the increase of impact velocity, the composite is ruptured mainly on center region where impact was applied and clamped positions. For the lateral damage shown in the left column, a higher impact velocity denotes a greater level of deflection. An impact velocity of 6 m/s renders the composites with a maximum deflection of 18 mm and completely destroys the sample. A lower impact velocity mainly causes resin breakage and interface debonding with cracks propagating along the yarn direction. Subsequently, the damage is demonstrated by fiber breakage as a result of increased impact velocity. Comparing failure morphologies at the front and back of CaCb with a specified impact velocity, the front exhibits a different failure mode from that of the back. For example, at an impact velocity of 4 m/s, the CaCb front damage is attributed to the compressed resin, whereas the back damage is ascribed to fiber breakage as the braided structure of the yarns created a bending surface that can bear tension rather than a compressive load.

**Figure 6:**
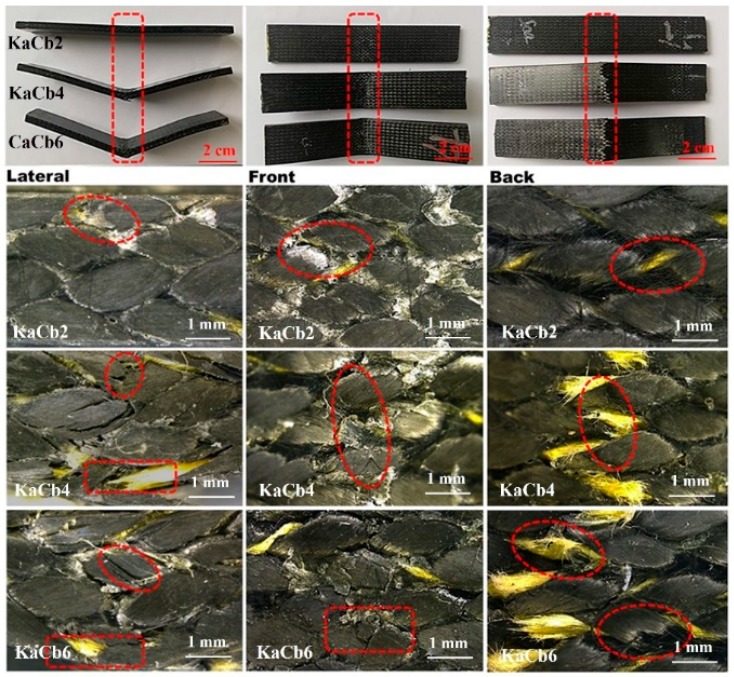
Damage morphology of 3D5d braided composite of KaCb series after impact with impact velocity of 2, 4 and 6 m/s from lateral, front and back views; subscripts 2, 4, 6 represent impact velocity 2, 4 and 6 m/s.

For 3D5d braided composite of KaCb series, the damage morphologies after impact are shown in Figure 6. KaCb comprises aramid fibers as the axial yarn and carbon fibers as the braiding yarn. Similar to CaCb, increasing the impact velocity causes more severe damage to the sample, however, the degree of damage relative to CaCb is reduced. After a 2 m/s impact, it is notable that no damage can be observed on the back of KaCb and aramid fibers hidden in carbon fiber start to show up in a twisted state due to the compression and shear of braiding yarns. The morphology after 4 m/s impact presents more severe resin crack and interface debonding, especially in back view of KaCb4, the fibrillation of aramid fiber can be clearly observed, which is resulted from the skin-core structure of the aramid fiber. At a higher impact velocity of 6 m/s, the fracture of carbon fibers is apparent. When subjected to higher compression loading during impact, the aramid fibers are extruded to the surface of composite, shown on the back view of KaCb6. Compared with the brittle fracture morphology of CaCb, the aramid fibers acting as the axial yarns help to maintain the integrated structure of composite after 6 m/s impact, proving that the hybrid effect strengthened the impact toughness and damage tolerance of composite.

**Figure 7:**
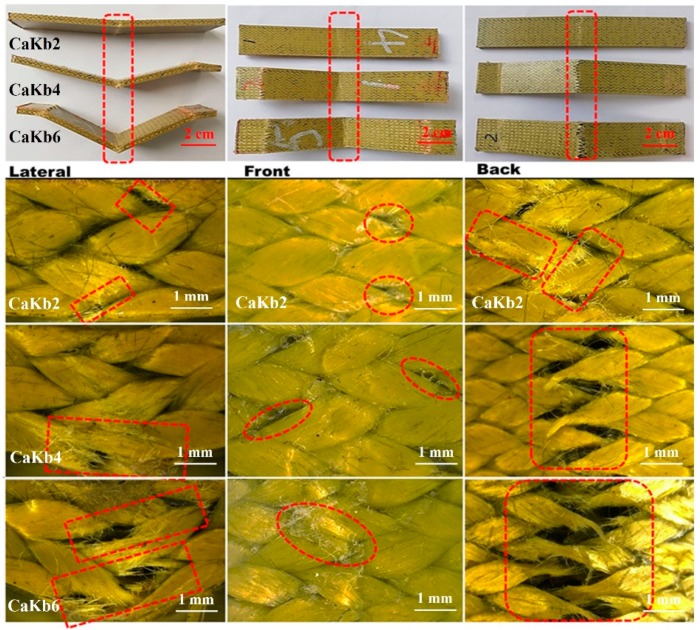
Damage morphology of 3D5d braided composite of CaKb series after impact with impact velocity of 2, 4 and 6 m/s from lateral, front and back views; subscripts 2, 4, 6 represent impact velocity 2, 4 and 6 m/s.

Due to carbon fibers acting as axial yarn and aramid fibers as braiding yarn, the damage morphology of CaKb mainly shows the features the aramid fiber fracture. From the front view, regardless of the impact velocity, due to the ductile characteristic of aramid fibers, almost no fiber breakage was observed except for some resin cracks (seen from middle column in Figure 7). However, the lateral and back views show server damage in the modes of fiber pullout and resin crack. Although the morphology of carbon fibers is not observed clearly from Figure 7, it can be inferred that the carbon fibers broke first under high impact velocity because of two factors: (1) The elongation of carbon fibers is lower than aramid fibers; (2) axial yarn bears more load than braiding yarn.

### 3.4. Ultrasonic Non-Destructive Analyses

**Figure 8:**
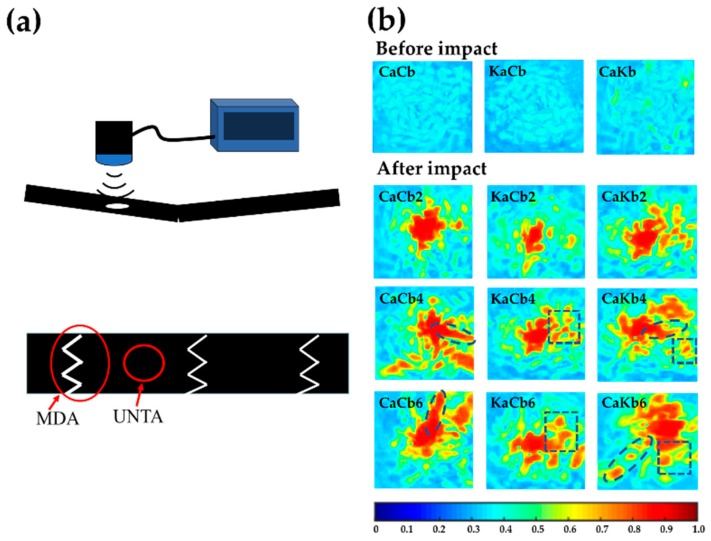
(**a**) Test area and (**b**) test results of 3D5d braided composites for ultrasonic non-destructive test.

An optical microscope can only be used to observe the macroscopic damage area of the 3D5d braided composites. For those regions with no apparent damage, ultrasonic nondestructive test can be an effective tool to qualitatively analyze the difference of internal damage. Since the architectures for three types of composite are kept the same, the C-scan difference can reflect the hybridization effect induced by the different types of fiber components. The ultrasonic nondestructive testing area was determined where no apparent damage is observed, as shown in Figure 8a. Given that the composites consisted of a 5d spatial interweaving structure, the ultrasonic reflection is formed with evenly distributed light blue pattern, as shown in images before impact in Figure 8b. It shows that increasing the impact velocity generates larger areas with warmer color, demonstrating more server damages have been formed. Moreover, a higher number of red regions indicate the higher level of interior damage. 

On the other hand, three types of composites demonstrate irregularly shaped damaged regions. An increase in the impact velocity produces strips for CaCb, blocks for KaCb, and strips and blocks for CaKb. The strips are present along the yarn direction, suggesting that damage occurs on the interface between the yarns and resin. The presence of an interfacial crack results in blocks, suggesting that local regions, such as those in resin, are destroyed. Based on the aforementioned analyses, three types of composites destroyed by the same impact loading demonstrate interior damage, which is highly dependent on the fiber component and interior alignments, especially for axial yarns, which bear more load than the braiding yarns. With carbon fibers as axial yarns, damage occurs on the interface between the yarns and resin. Using aramid fibers as axial yarns leads to resin failure, proving that aramid fibers exhibit greater interfacial compatibility than carbon fibers.

### 3.5. Impact Responsive Frequency Analyses

To investigate the relationship between the load curves and failure mode of 3D5d braided composites, we processed the load signals with Fourier transform and wavelet transform to obtain the frequency and time domains, respectively. The damage mechanism hidden in the time–load curves can be examined using the time-frequency analysis. Frequency domain analysis was used to obtain the damage and mode changes, and time domain analysis was employed to obtain the degree of damage [23].

#### 3.5.1. Frequency Domain Analyses via Fourier Transform

The load signals were converted using fast Fourier transform. The Fourier transform for non-periodic continuous signal is as follows:

(3) $$F\left(w\right)=\int_{-\infty }^{+\infty }f\left(t\right)e^{-\mathrm{jwt}}\mathrm{dt}$$

where w, t, and $e^{-\mathrm{jwt}}$ indicate the frequency, length of time, and complex function, respectively.

The discrete sampling value x(*n*) of continuous signals can be obtained in a real manipulating system. Therefore, the discrete signal was used to compute the frequency spectrum of signal x(*t*). The discrete Fourier transform of a discrete signal of finite length x(*n*), (*n* = 0, 1…., N − 1) is defined as follows:

(4) $$X\left(k\right)=\overset{N-1}{\underset{n=0}{\sum }}x\left(n\right)W_{N}^{\mathrm{kn}},k=0,1,\ldots \ldots ,N.{\;W}_{N}=e^{-j\frac{2\pi }{N}}\;$$

where *n*, *k*, and *N* refer to the time index, frequency index, and finite-length discrete time series, respectively.

**Figure 9:**
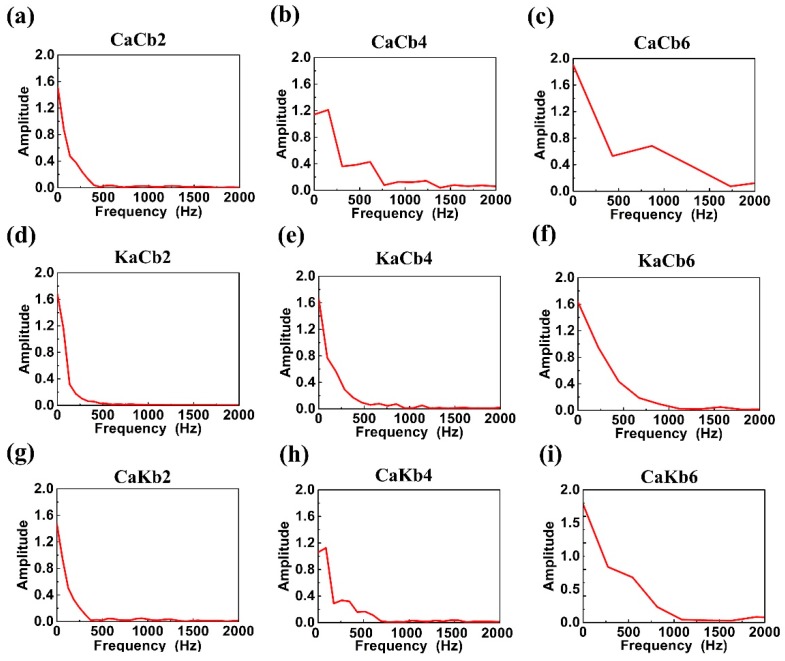
Impact responsive frequency distribution of 3D5d braided composites. The three rows represent the sample types of (**a**–**c**) CaCb, (**d**–**f**) KaCb, and (**g**–**i**) CaKb, whereas the three columns represent the impact velocities of (**a**,**d**,**g**) 2 m/s, (**b**,**e**,**h**) 4 m/s, and (**c**,**f**,**i**) 6 m/s.

Figure 9 shows the impact responsive frequency distribution plots of 3D5d braided composites. A high impact velocity positively influences the frequency domain range of CaCb, KaCb, and CaKb. Different frequency domains exhibit their corresponding failure modes, indicating that failure mode is dependent on the increase in impact velocity, which was also found in Zhang et al.′s work [24]. At 2 m/s impact velocity, the frequency domain reaches 200 Hz. When combined with fracture morphologies, it suggests that failure mode occurs due to resin cracks in 0–200 Hz. At 4 m/s impact velocity, the frequency domain is expanded to 500 Hz, suggesting that both resin crack and fiber breakage appear, and failure mode was due to fiber breakage [23,24]. Figure 9b,e,h show that KaCb demonstrates a sleek and dense frequency domain distribution curve. Conversely, CaCb exhibits a folded line, indicating a sporadic distribution of frequency domain, especially at an impact velocity of 6 m/s. KaCb and CaCb show the opposite failure mechanisms, which is ascribed to the breakage mechanism of carbon and aramid fibers and their interfaces with resin. At 6 m/s impact velocity, the impact wave is observed at 1000 Hz, and the major failure mode results from fiber breakage, which can also be proved from Figure 5, Figure 6 and Figure 7. Resin and fibers are ruptured differently at a high impact velocity, causing differences in the domain ranges of failure mode between 4 and 6 m/s. 

#### 3.5.2. Time Domain Analyses via Wavelet Transform

In order to better analyze the degree of damage under low-velocity impact, the received response signals are normalized and analyzed in time domain [25,26,27]. Time-load signals were converted and then dissected into five layers using the dymeyer of Matlab (MathWorks, Natick, MA, USA), thereby obtaining an approximate signal and five detailed signals. The Meyer wavelet equation is as follows:

(5) $$\varphi =\{\begin{array}{cc} {\left(2\pi \right)}^{\frac{1}{2}} & \;(|\xi |\leq \frac{2\pi }{3}),\\ {\left(2\pi \right)}^{\frac{-1}{2}}\cos\left[\frac{\pi }{2}\nu \left(\frac{3}{2\pi }|\xi |-1\right)\right] & \;(\frac{2\pi }{3}\leq \left|\xi \right|\leq \frac{4\pi }{3})\\ \;0 & \;(\mathrm{others}) \end{array}$$

which $\nu \left(x\right)=\left\{\begin{array}{c} 0\;\;\left(x\leq 0\right)\\ 1\;\;\left(x\geq 1\right) \end{array}\right\},\nu \left(x\right)+\nu \left(1-x\right)=1.$

**Figure 10:**
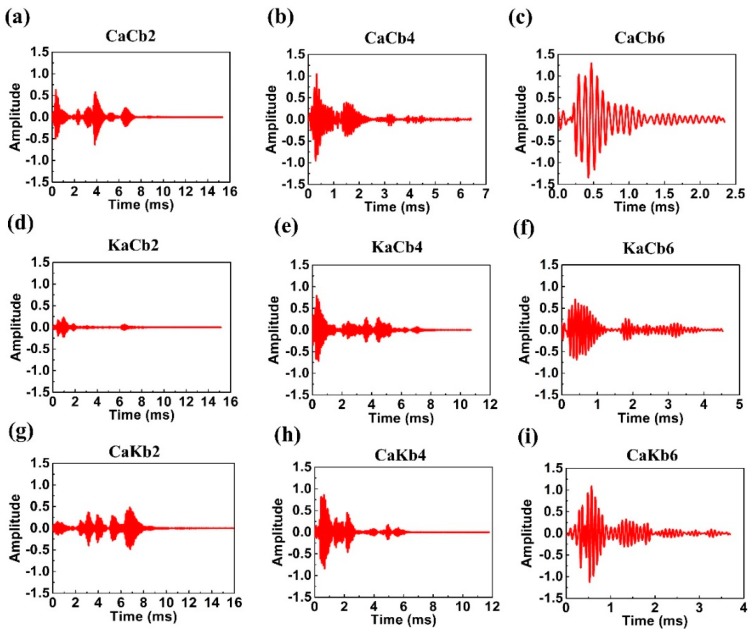
Detailed impact load signals of 3D5d braided composites. The three rows represent the samples of (**a–c**) CaCb, (**d**–**f**) KaCb, and (**g**–**i**) CaKb, whereas the three columns represent the impact velocities of (**a**,**d**,**g**) 2 m/s, (**b**,**e**,**h**) 4 m/s, and (**c**,**f**,**i**) 6 m/s.

The original data were then processed with wavelet analysis to obtain approximate and detailed signals. Based on the results in Section 3.5.1, the impact rupture waves were primarily noted at low frequency, and the detailed signals obtained from low frequencies were used. Based on the detailed signals, different impact–time points correspond to different amplitudes. The differences in amplitude can be considered to be caused by different structures. Therefore, detailed signals can be used to analyze the degree of damage during the whole corresponsive process [27]. Figure 10 shows the detailed signal of an impact load chart, where the detailed signals demonstrate the increasing amplitude of the three composite types as a result of increasing impact velocity, indicating a high level of damage. At 2 m/s impact velocity, two larger amplitudes of CaCb2 and small amplitudes of KaCb2 and CaKb2 occur in the initial stage and are continually magnified until the test completion. The initial amplitudes are related to the stiffness of materials. CaCb exhibits a greater stiffness than the other two groups and demonstrates a greater level of damage at an impact velocity of 4 m/s. The different patterns in amplitudes show that no damage was made to KaCa, whereas continual damage occurred during the whole test for CaKb.

At 4 m/s impact velocity, CaCb4, KaCb4, and CaKb4 demonstrate significant level of damage in the initial stage, whereas CaCb4 and CaKb4 exhibit complete ruptures at 3 ms. However, the rupture of KaCb4 continues until 5 ms. Finally, when the impact velocity was 6 m/s, the number of vibrations decrease, indicating the simultaneous presence of damage and damage evolution. CaCb6 demonstrates the greatest amplitude, followed by CaKb6 and KaCb6. Regardless of whether aramid fibers are used as braiding yarns or axial yarns, large amplitudes are absent in the initial stage, suggesting that the presence of aramid fibers decreases the level of initial damage. Furthermore, when using axial yarns, aramid fibers effectively mitigates the level of damage during damage evolution.

## 4. Conclusions

Damage morphology analyses confirmed that axial yarns bore the impact force for CaCb, KaCb, and CaKb. With aramid fibers as axial yarns, the 3D5d braided composites showed notable impact resistance. The extensive interface of aramid fibers also provided synergistic effect for the composites to provide a functioning space for braiding yarns (i.e., carbon fibers). Ultrasonic nondestructive testing was conducted to examine the interior damage. The difference in the interfaces between carbon fibers/aramid fibers and resin resulted in the different shapes of interior rupture cracks. CaCb exhibited strip-liked cracks due to the delamination between carbon fibers and resin, KaCb demonstrated block-like cracks due to resin failure, and CaKb showed cracks at both shapes. Therefore, with the same impact load, interior damage was dependent on the materials and their interior location. The load–displacement curve analyses show that the 3D5d braided composites can effectively stop crack propagation, especially in CaCb, which demonstrated the highest efficiency and maintained stable crack propagation. Energy–displacement curves showed that when the total rupture status was reached, KaCb absorbed the maximum impact energy, which was primarily used to stabilize crack propagation and achieve the greatest ductility. In energy distribution pattern analyses, KaCb showed an optimal impact resistance because external loading was applied and then transmitted to the exterior carbon fiber braiding yarns. Carbon fibers with high stiffness can rapidly dissipate the load to the surroundings, reducing the local force. Due to the superior toughness of aramid fibers, the load was transmitted to axial yarns (i.e., aramid fibers) and the whole load delivery process was an energy absorption process. According to the load signals analysis, a high impact velocity enlarged the frequency domain range of the load. Comparison of the frequency domain and failure modes demonstrated that resin crack and fiber breakage occurred at 0–200 and 200–1000 Hz, respectively. Wavelet transform was used to analyze the differences in the level of damage of 3D5d braided composites. Aramid fibers were used as either axial yarns or braiding yarns, effectively decreasing the level of initial damage. In particular, when used as axial yarns, aramid fibers effectively mitigate the level of damage during damage evolution. 

## Figures and Tables

**Table 1:** Raw material parameters.

Materials	Type	Strength (MPa)	Modulus (GPa)	Density (g/cm^3^)	Elongation (%)
Carbon fiber	T300-3K	3530	230	1.76	1.5
Aramid fiber	Kevlar49	2923	175	1.44	2.8
Resin	TDE-86	80	3.5	1.13	4.0

**Table 2:** Composite specifications.

Type	Axial	Braid	Pick Length (mm)	Pick Width (mm)	Yarn Volume Content (%)
CaCb	T300-3K	T300-3K	3 ± 0.1	2.2 ± 0.1	T300-3K: 53%
KaCb	Kevlar49	T300-3K	3 ± 0.1	2.4 ± 0.1	Kevlar49: 11%; T300-3K: 43%
CaKb	T300-3K	Kevlar49	3 ± 0.1	2.2 ± 0.1	Kevlar49: 43%; T300-3K: 11%
